# Biosynthesis of pinene in purple non-sulfur photosynthetic bacteria

**DOI:** 10.1186/s12934-021-01591-6

**Published:** 2021-05-17

**Authors:** Xiaomin Wu, Guang Ma, Chuanyang Liu, Xin-yuan Qiu, Lu Min, Jingyu Kuang, Lingyun Zhu

**Affiliations:** 1 Department of Biology and Chemistry, College of Liberal Arts and Sciences, National University of Defense Technology, Changsha, 410073 Hunan China; 2 China Astronaut Research and Training Center, Beijing, 100094 China

**Keywords:** Purple non-sulfur photosynthetic bacteria, Metabolic-engineered, Pinene production, Heterologous

## Abstract

**Background:**

Pinene is a monoterpene, that is used in the manufacture of fragrances, insecticide, fine chemicals, and renewable fuels. Production of pinene by metabolic-engineered microorganisms is a sustainable method. Purple non-sulfur photosynthetic bacteria belong to photosynthetic chassis that are widely used to synthesize natural chemicals. To date, researches on the synthesis of pinene by purple non-sulfur photosynthetic bacteria has not been reported, leaving the potential of purple non-sulfur photosynthetic bacteria synthesizing pinene unexplored.

**Results:**

*Rhodobacter sphaeroides* strain was applied as a model and engineered to express the fusion protein of heterologous geranyl diphosphate synthase (GPPS) and pinene synthase (PS), hence achieving pinene production. The reaction condition of pinene production was optimized and 97.51 μg/L of pinene was yielded. Then, genes of 1-deoxy-d-xylulose 5-phosphate synthase, 1-deoxy-d-xylulose 5-phosphate reductoisomerase and isopentenyl diphosphate isomerase were overexpressed, and the ribosome binding site of GPPS-PS mRNA was optimized, improving pinene titer to 539.84 μg/L.

**Conclusions:**

In this paper, through heterologous expression of GPPS-PS, pinene was successfully produced in *R. sphaeroides*, and pinene production was greatly improved by optimizing the expression of key enzymes. This is the first report on pinene produce by purple non-sulfur photosynthetic bacteria, which expands the availability of photosynthetic chassis for pinene production.

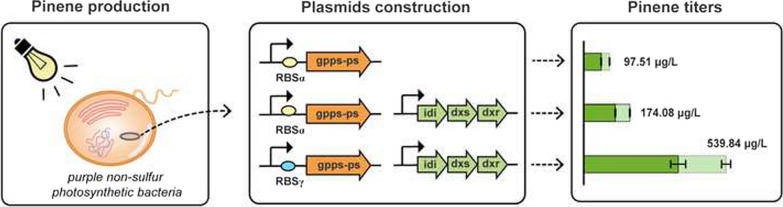

**Supplementary Information:**

The online version contains supplementary material available at 10.1186/s12934-021-01591-6.

## Background

**Fig. 1:**
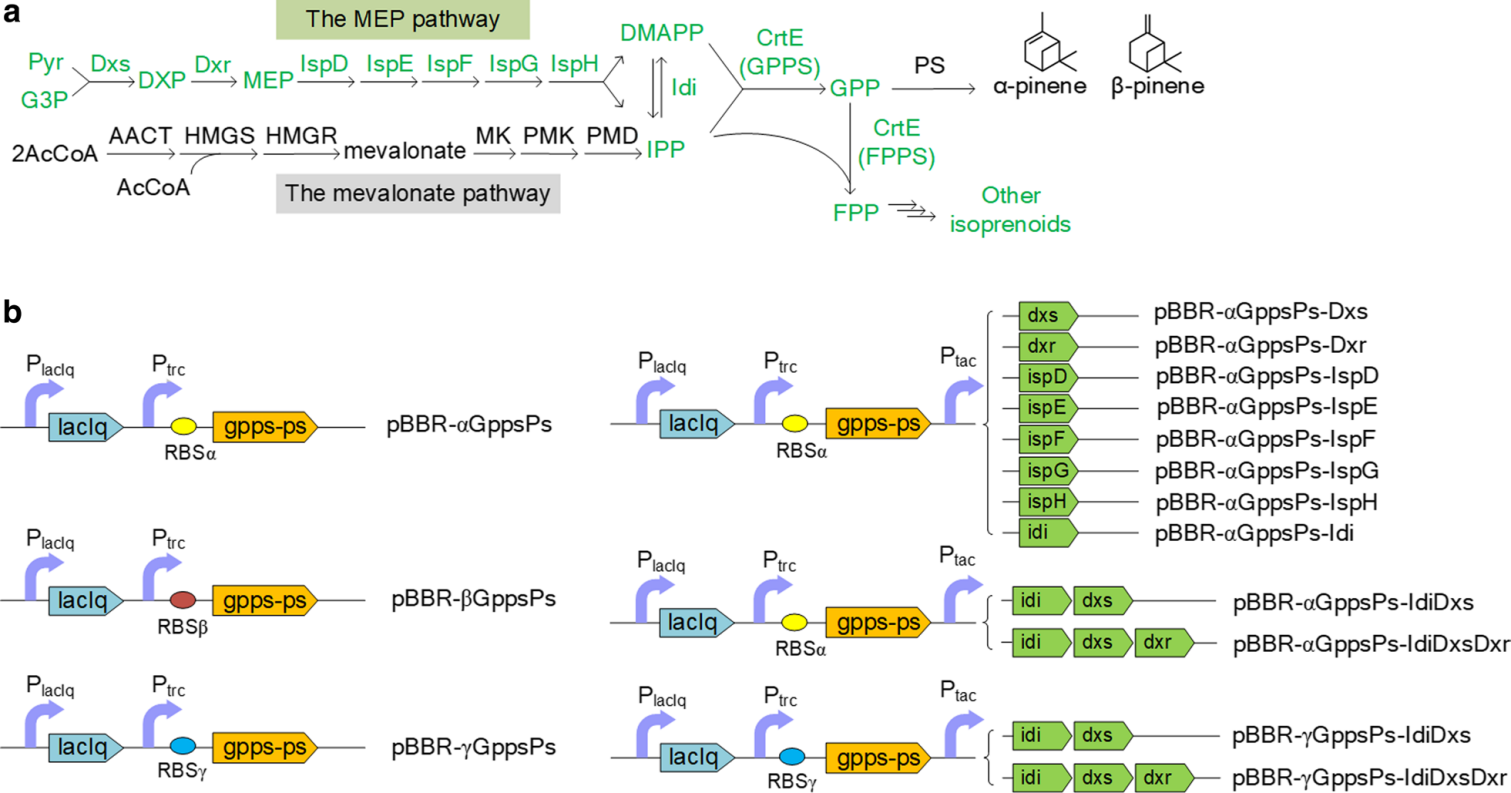
The schematic of the terpene synthetic pathway and the functional operons of plasmids used in this study. **a** Biosynthetic pathway of isoprenoids. The intrinsic terpene biosynthesis of photosynthetic bacteria is shown in green. **b** The functional operons of plasmids used for the biosynthesis of pinene in *R. sphaeroides*. Pyr, pyruvate; G3P, glyceraldehyde-3-phosphate; DXP, 1-deoxy-d-xylulose 5-phosphate; MEP, 2C-methyl-d-erythritol 4-phosphate; DMAPP, dimethylallyl pyrophosphate; IPP, isopentenyl pyrophosphate; GPP, geranyl pyrophosphate; FPP, farnesyl pyrophosphate; Dxs, 1-deoxy-d-xylulose 5-phosphate synthase; Dxr, 1-deoxy-d-xylulose 5-phosphate reductase; IspD, 2C-methyl-d-erythritol 4-phosphate cytidylyltransferase; IspE, 4-diphosphocytidyl-2C-methyl-d-erythritol kinase; IspF, 2C-methyl-d-erythritol 2,4-cyclodiphosphate synthase; IspG, 2C-methyl-d-erythritol 2,4-cyclodiphosphate reductase; IspH, 4-hydroxyl-3-methylbut-2-enyl diphosphate reductase; Idi, isopentenyl diphosphate isomerase; CrtE, prenyltransferase, having both geranyl diphosphate synthase (GPPS) and farnesyl diphosphate synthase (FPPS) activity; PS, pinene synthase; AcCoA, acetyl-CoA; AACT, acetoacetyl-CoA thiolase; HMGS, 3-hydroxy-3-methyl-glutaryl-CoA synthase; HMGR, 3-hydroxy-3-methyl-glutaryl-CoA reductase; MK, mevalonate kinase; PMK, phosphomevalonate kinase; PMD, diphosphomevalonate decarboxylase

Terpenes are natural compounds that can be used as fragrances, flavorings agents, medicines and potential biofuels [1]. Terpenes are isoprenoids, synthesized from isopentenyl intermediates by corresponding terpene synthases, and these isopentenyl intermediates are condensed from C5 isoprene units, namely isopentenyl diphosphate (IPP) and dimethylallyl diphosphate (DMAPP) [2]. IPP and DMAPP are common building blocks of isoprenoids, which are synthesized from three molecules of acetyl-CoA via the mevalonate pathway or from pyruvate and glyceraldehyde-3-phosphate via the 2C-methyl-d-erythritol 4-phosphate (MEP) pathway shown in Fig. 1a [3]. These two isoprenoid pathways exist in different species. The mevalonate pathway is dominant in eukaryotes, archaea, and cytoplasm of higher plants, and the MEP pathway is found in most bacteria, green algae, and chloroplasts of higher plants [2, 3].

Pinene (C10) is a monoterpene, which comes in the form of α-pinene and β-pinene. They can be precursors of different aroma compounds used in cosmetic, food and medicine industry [4]. Besides, they both have potential as feedstocks for high-density renewable fuels, for pinene dimers contain high volumetric energy [5]. Pinene is generally synthesized in plants, converted from geranyl diphosphate (GPP) by pinene synthase (PS), while GPP is synthesized by geranyl diphosphate synthase (GPPS) from condensing IPP and DMAPP shown in Fig. 1a [6]. In addition, the pinene could also be produced by microorganisms through metabolic engineering, which is thought to be a sustainable method [7]. Yang et al., constructed the mevalonate pathway, GPPS and PS genes in *E.coli*, which led to pinene production (5.44 mg/L) under shake-flask conditions [8]. Then, pinene yield in *E.coli* was further improved by introducing GPPS-PS protein fusions [6] and evolved geranyl diphosphate synthase and pinene synthase with high activity [9, 10]. With the expression of pinene synthase, pinene can also be produced in other microorganisms, e.g. Cyanobacterial [9] and *Corynebacterium glutamicum* [11].

The purple non-sulfur photosynthetic bacteria are non-oxygen-producing bacteria, with a variety of metabolic modes that enable them to grow under phototrophic conditions or in darkness conditions by respiration, fermentation, or chemolithotrophy [12]. The diversity of metabolism allows purple non-sulfur photosynthetic bacteria to use a variety of carbon sources [13], and they have been studied as phototrophic platform organisms for valuable chemicals production [14]. For example, purple non-sulfur photosynthetic bacteria have been used to produce poly-β-hydroxyalkanoates [15], membrane proteins [16], hydrogen gas [17], and carotenoids [18]. Moreover, purple non-sulfur photosynthetic bacteria have abundant inner membrane system, which act as a container for hydrophobic metabolites [14]. They have been used to study the synthesis of sesquiterpenes, triterpenes, tetraterpenes, etc., through the endogenous MEP pathway or heterologous mevalonate pathway [14, 19, 20]. However, researches on the synthesis of pinene by purple non-sulfur photosynthetic bacteria have not been reported, leaving the potential of purple non-sulfur photosynthetic bacteria in pinene production undetermined.

*Rhodobacter sphaeroides* is a kind of widely studied purple non-sulfur photosynthetic bacteria, which is applied as a bio-factory to synthesize farnesol [21], coenzyme Q_10_ [22], lycopene [20] and valencene [23]. In this paper, *R. sphaeroides* strain was used as a model and a fusion protein of GPPS and PS was introduced to it to allow pinene synthesis. Through genetic engineering and reaction conditions optimization, the potential of pinene production in purple non-sulfur photosynthetic bacteria was tested.

## Results

### Pinene production using protein fusions

Purple non-sulfur photosynthetic bacteria use the endogenous MEP pathway to produce IPP and DMAPP, which are further catalyzed by CrtE to synthesize GPP. Then, GPP can be catalyzed to pinene by introducing an exogenous PS gene, as shown in Fig. 1a. However, CrtE have both GPP synthase and farnesyl diphosphate (FPP) synthase activity. Thus, CrtE can convert IPP and DMAPP to GPP, then to FPP. As an intermediate, GPP is more readily catalyzed to FPP, instead of generating monoterpenes, e.g. pinene [24].

**Fig. 2:**
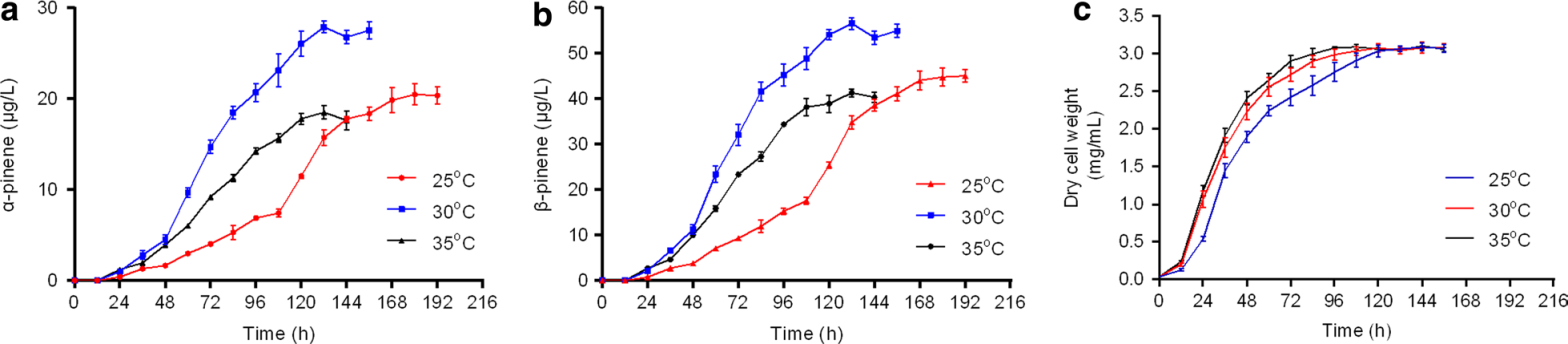
Pinene production and dry cell weight of *R. sphaeroides* harboring pBBR-αGppsPs plasmid over time at various temperatures with the induction of 3 µM IPTG. **a** The titers of α-pinene. **b** The titers of β-pinene. **c** Dry cell weight of *R. sphaeroides*:pBBR-αGppsPs. Errors indicate s.d. (n = 3)

To elevate the metabolic flux from GPP to pinene, a rational strategy is to construct a fusion protein of GPPS and PS to make it easier for GPP to enter PS active site from GPPS active site [6, 9]. In this study, a pBBR-αGppsPs plasmid was constructed, in which a fusion gene of *Abies grandis* GPPS and PS was expressed under the strong trc promoter and with the ribosome binding site of RBSα. The pBBR-αGppsPs plasmid was transferred to *R. sphaeroides* by conjugation and the final strain (*R. sphaeroides*:pBBR-αGppsPs) was grown under anaerobic conditions with illumination to produce pinene. Pinene titers were detected over time at various temperatures to obtain the optimal culturing temperature and duration. As shown in Fig. 2, improved production of α-pinene and β-pinene could be observed under 30℃ comparing to 25 and 35℃, while the growth rates of the strain remained similar under all these temperatures. These results indicated that maintaining the temperature at 30℃ was more suitable for pinene production. It was observed the pinene titers gradually increasing to maximum and then reached plateau after culturing for 132 h at 30℃, so the optimal culturing duration was 132 h.

**Fig. 3:**
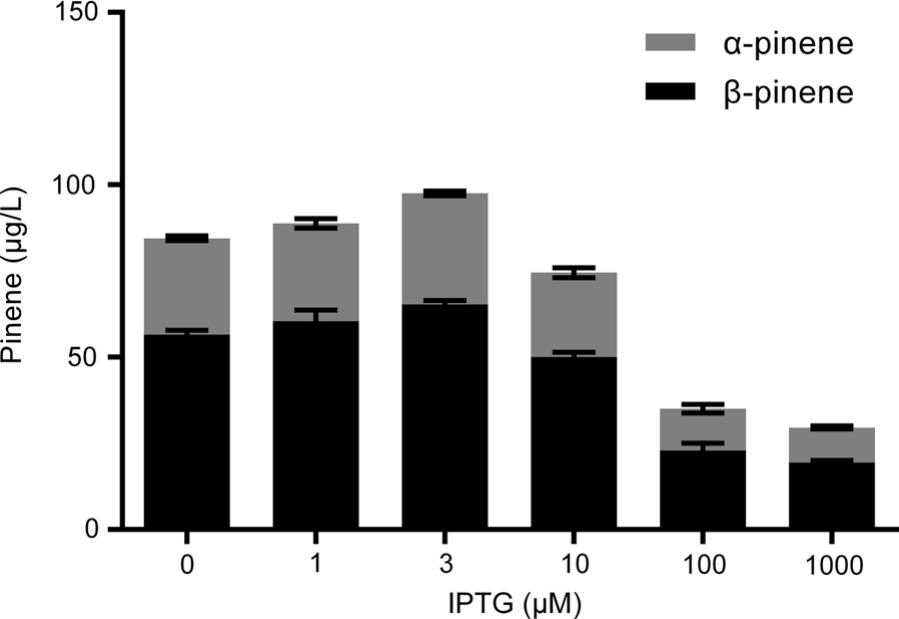
Pinene production at various IPTG concentrations by *R. sphaeroides* harboring pBBR-αGppsPs plasmid. Errors indicate s.d. (n = 3)

As the transcription of *gpps-ps* was induced by IPTG, we tested the effect of IPTG concentrations on pinene production. Figure 3 showed pinene production using GPPS-PS fusion protein at various IPTG concentrations, ranging from 0 to 1000 μM. At the IPTG concentration of 3 μM, the highest pinene titer was obtained, which was 97.51 μg/L in total (32.17 μg/L α-pinene and 65.34 μg/L β-pinene). When IPTG concentration was higher than 10 μM, the pinene titers were much lower than the value at the IPTG concentration of 3 μM. This suggests that the increase in IPTG concentration will inhibit the synthesis of pinene in *R. sphaeroides* which is consistent with reduced cell growth under high IPTG concentrations (Additional file 1: Fig. S1). Thus, in the subsequent pinene production reaction, the concentration of IPTG was set to 3 μM.

### Improving pinene production by overexpression of key genes

As most genes in isoprenoid pathway are expressed at low level under normal growth conditions, the common method is to increase the expression of key genes to improve the metabolic flux [25]. The genes coded for MEP pathway enzymes were studied and four genes (*dxs*, *idi*, *ispD* and *ispF*) were deemed to be the rate-limiting in *E. coli* [25, 26]. Lu et.al overexpressed *dxs*, *dxr*, *ispD* and *idi* simultaneously and increased Q_10_ yield by two folds in *R. sphaeroides* [22]. However, there is limited information about the MEP pathway in purple non-sulfur photosynthetic bacteria and the key genes were not confirmed by experiments.

In this study, the genes in MEP pathway were overexpressed and pinene production was tested, respectively, in order to determine the key genes. Then, the key genes were co-expressed to increase metabolic flux towards IPP and DMAPP.

**Fig. 4:**
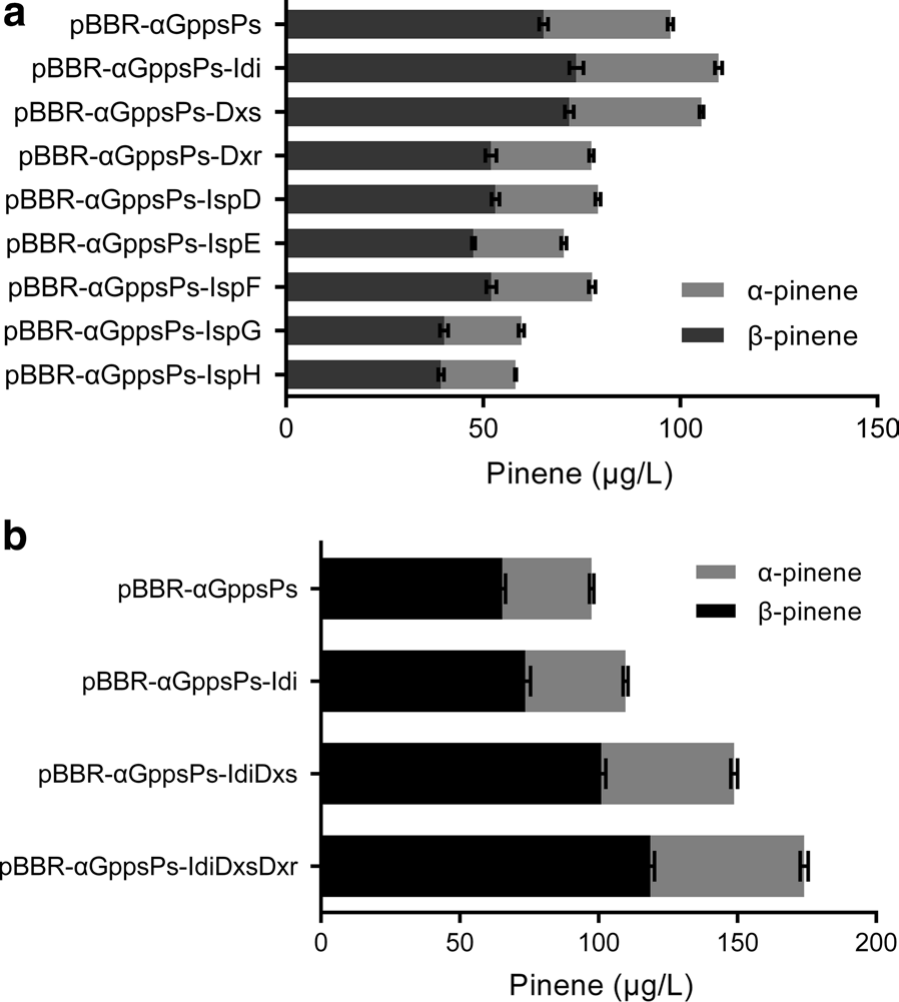
Pinene production by *R. sphaeroides* harboring plasmids derived from pBBR-αGppsPs with overexpression of *dxs*, *dxr*, *ispD*, *ispE*, *ispF*, *ispG*, *ispH* and *idi* genes (**a**), and genes of rate-limiting enzymes (**b**), respectively. Errors indicate s.d. (n = 3)

Eight plasmids with the expression of *dxs*, *dxr*, *ispD*, *ispE*, *ispF*, *ispG*, *ispH* and *idi* were constructed based on pBBR-αGppsPs (Fig. 1b), and then transferred to *R. sphaeroides*, respectively. Figure 4a showed pinene production with overexpression of these genes. Pinene titers were improved to 105.34 and 109.73 μg/L with overexpression of *dxs* and *idi*, respectively, indicating that Dxs and Idi are rate-limiting enzymes of MEP pathway in *R. sphaeroids*, which is consistent with the reports that Dxs and Idi enhanced carbon flux of isoprenoid biosynthesis in *E.coli* [27, 28]. We therefore co-expressed *idi* and *dxs* together (pBBR-αGppsPs-IdiDxs) and further improved pinene production to 148.83 μg/L (Fig. 4b).

In contrast, overexpression of *dxr*, *ispD*, *ispE*, *ispF*, *ispG* and *ispH*, respectively, resulted in a decrease in pinene production. This suggests overexpression of these genes might cause metabolic burden or toxicity.

Considering that Dxr was reported to be a key enzyme in *E.coli* [29], *dxr* was overexpressed with *idi* and *dxs* together (pBBR-αGppsPs-IdiDxsDxr) to improve pinene production. As shown in Fig. 4b, the strain overexpressing *idi*, *dxs* and *dxr* produced a higher amount of pinene (174.08 μg/L) than the one overexpressing *idi dxs* combination. This result indicates that Dxr turns into a rate-limiting enzyme, when the primary rate-limiting steps are released, and overexpression of *dxr* may further improve metabolic flux. In summary, overexpressing *idi*, *dxs* and *dxr* simultaneously causes a great increase of pinene output.

### Improving pinene production by altering RBS of GPPS-PS mRNA

**Fig. 5:**
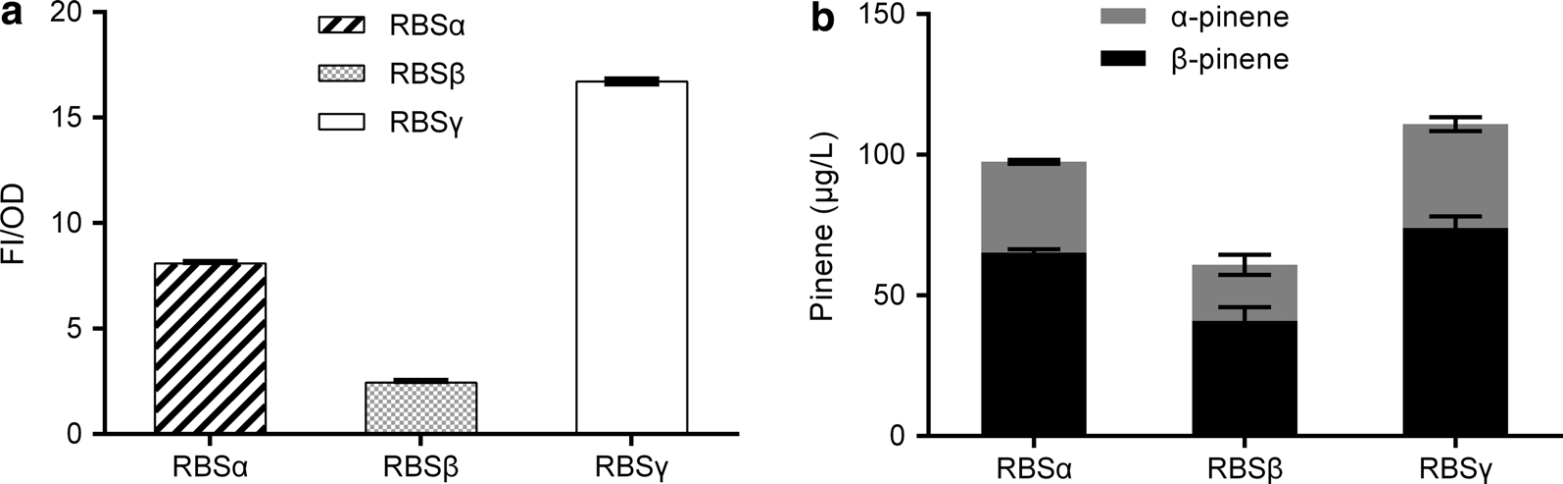
Test of various RBS. **a** The fluorescent intensity of GFP with various RBS. **b** Pinene production via GPPS-PS fusion protein with various RBS. Errors indicate s.d. (n = 3)

The expression level of GPPS-PS protein can be altered by changing ribosome binding site, as the ribosome binding site plays an important role in protein translation. Therefore, we considered increasing pinene production by optimizing ribosome binding site. Two standard RBS sequences (RBSβ and RBSγ) as well as RBSα, were all taken from iGEM toolbox. The strength of RBSα, RBSβ, and RBSγ were tested with GFP as the reporter protein. As shown in Fig. 5a, the fluorescent intensity of GFP is stronger with RBSγ compared to RBSα, but is weaker with RBSβ. Then, the ribosome binding site of GPPS-PS mRNA (RBSα) was replaced by a stronger RBS (RBSγ) and a weaker RBS (RBSβ), and two plasmids (pBBR-βGppsPs, pBBR-γGppsPs) were transferred to *R.sphaeroides*, respectively, then the resulting pinene titers were compared. Figure 5b shows that the pinene production can be improved by using a stronger RBS, but decreased with a weaker RBS. Thus, overexpression of GPPS-PS protein could increase pinene production, which sμggests the metabolic fluxes of downstream pathway might be inadequate in the strain harboring pBBR-αGppsPs.

**Fig. 6:**
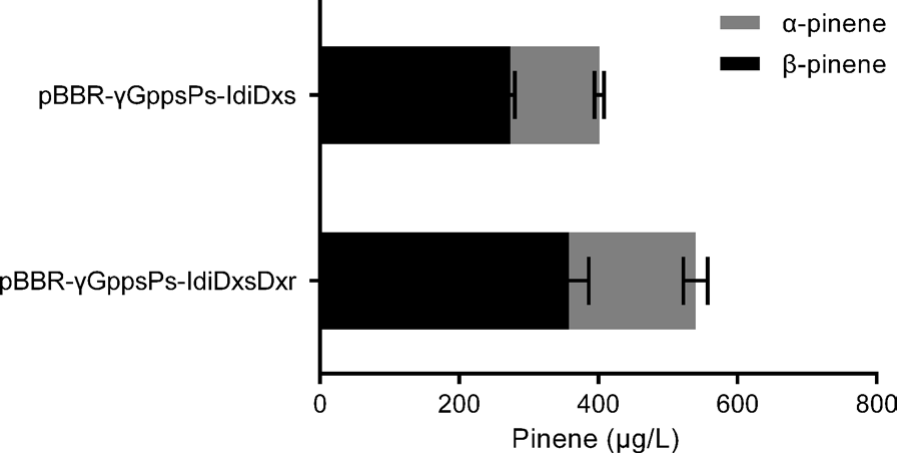
Pinene production by *R. sphaeroides* harboring plasmids derived from pBBR-γGppsPs with overexpression of genes of rate-limiting enzymes. Errors indicate s.d. (n = 3)

Therefore, RBSα was replaced with RBSγ, and pBBR-γGppsPs-IdiDxs and pBBR-γGppsPs-IdiDxsDxr were constructed and transferred to *R.sphaeroides*, respectively. As shown in Fig. 6, overexpresssing *idi* and *dxs* increased the production of pinene to 401.43 μg/L and overexpressing *idi*, *dxs* and *dxr* together further improved the yield of pinene to 539.84 μg/L, which was 5.54 folds compared to *R. sphaeroides*:pBBR-αGppsPs strain. Thus, overexpression of key enzymes of the MEP pathway and the downstream pathways simultaneously can substantially increase pinene production.

## Discussion

To date, heterogeneous terpenoids production were mainly achieved in yeast [30] and *E. coli* [31]. Researches on the synthesis of pinene in other microorganisms were limited. Previous study used cyanobacteria to synthesize pinene with an engineered pinene synthase under phototrophic conditions and reached 80 μg/L pinene production [9]. In our work, we showed 539.84 μg/L pinene production in engineered *R. sphaeroides* under phototrophic conditions. Thus, photosynthetic bacteria can be used as an alternative photosynthetic chassis.

Compared with *E.coli*, the production of pinene in phototrophic microorganisms is much lower. In fact, phototrophic microorganisms synthesize a large amount of compounds with IPP and DMAPP as precursors, such as carotenoids [32], coenzyme Q_10_ [22], lycopene [20], through the MEP pathway and downstream pathways. Researchers improved the yield of target products by weakening some competitive pathway. For example, Zhu YQ increases the production of coenzyme Q_10_ by reducing the synthesis of carotenoids [33]. For pinene production, we can reduce the synthesis of the above metabolites to guide more metabolism flux to the direction of pinene synthesis. In addition, the growth rates of phototrophic microorganisms are much slower than *E. coli*. Increasing the growth rate is significant for phototrophic microorganisms to be developed into bioproduction platforms. Researchers have constructed fast-growing cyanobacteria by expressing key rate-limiting enzymes, with a doubling time of 2–3 h [34]. Therefore, understanding the rate-limiting enzymes of purple non-sulfur photosynthetic bacteria growth is of great significance for improving the growth rate.

In addition to optimizing the endogenous MEP pathway, introduction of a heterogeneous mevalonate metabolic pathway is also a common method [35]. We tried to use plasmids to introduce the mevalonate pathway genes with gpps-ps gene simultaneously, but the pinene production was severely reduced (data not shown). A similar phenomenon was also found in the synthesis of patchoulol by *R. capsulatus* [14]. When a plasmid was used to express the mevalonate pathway and multiple other genes simultaneously, patchoulol could not be detected [14]. The possible reason might be that the overexpression of mevalonate pathway genes brought great metabolic burden and growth pressure to the host. However, when the exogenous mevalonate pathway was recombined into the genome, patchoulol production can be increased [14]. Therefore, an effective way to introduce the heterogeneous mevalonate pathway is to recombine it into the genome. In addition, improving the activity of pinene synthase can greatly increase the output of pinene [9]. It is also very important to further screen for pinene synthase with high activity.

## Conclusions

In our work, we successfully synthesized pinene in purple non-sulfur photosynthetic bacteria for the first time by heterologous expressing a fusion protein of geranyl diphosphate synthase and pinene synthase. The optimal temperature and IPTG concentration for pinene synthesis were 30 °C and 3 μM, respectively, and the optimal duration for the reaction was 132 h. Using the appropriate RBS and overexpressing *idi*, *dxs* and *dxr* simultaneously, the pinene titer can be increased by 5.54 folds to 539.84 μg/L. However, for the synthesis of pinene in purple non-sulfur photosynthetic bacteria, there is still a lot of work that can be done, such as expressing and optimizing a heterogeneous mevalonate pathway, screening for highly active pinene synthase, weakening the competitive metabolic pathway, and increasing the growth rate of purple non-sulfur photosynthetic bacteria.

## Methods

### Microorganisms and growth conditions

*E.coli* DH5α and *E.coli* S17-1 were applied for plasmid construction and di-parental conjugation, respectively. *R. sphaeroids* 2.4.1 (ATCC17023) was used for pinene production. *E. coli* strains were grown at 37 °C in Luria–Bertani medium consisting of yeast extract (5 g/L), tryptone (10 g/L), NaCl (10 g/L). For conventional cultivation and di-parental conjugation, *R. sphaeroides* strains were cultivated in modified Sistrom's medium [36] with or without agar. For pinene production, *R. sphaeroides* was grown photoheterotrophically with light intensity of ~ 4000 lx in modified Sistrom's medium supplied with glucose (30 g/L) as carbon source. The pH of the Sistrom's medium was maintained at 6.8–7.0 with 20 mM of phosphate buffer (final concentration). In addition, the media were supplemented with 50 mg/ L of kanamycin (Km) for both *R. sphaeroides* and *E.coli* when necessary.

### Plasmids and ribosome binding sites

The plasmids and ribosome binding sites (RBSs) used in this study are listed in Table 1. All the plasmids were constructed based on pBBR-trcGFP-lacIq and pBBR-tacGFP, which were both derived from broad host plasmid pBBR1MCS-2 with kanamycin resistance. The *gpps* and *ps* gene were both from *Abies grandis* [6], and the *gpps-ps* fusion gene were synthesized by Sangon Biotech (Shanghai) Co. Ltd., after codon optimization. The *idi*, *dxs*, *dxr*, *ispD*, *ispE*, *ispF*, *ispG* and *ispH* genes were all cloned from the genome of *Rhodobacter sphaeroides* 2.4.1. All the information for plasmids construction, including the method, all the primers and sequences of synthetic *gpps-ps* gene, pBBR-trcGFP-lacIq and pBBR-tacGFP are available from Mendeley Data [37]. The restriction enzymes *XbaI* and *EcoRI* used to linearize plasmids were purchased from New England Biolabs. The In-Fusion PCR cloning system (Clontech) was applied to ligate gene fragments and plasmid backbone in order to construct new plasmid.

**Table 1:** Plasmids and RBSs used in this study

Name	Description	References
pBBR-αGppsPs	Ptrc:*lacO*-RBSα-*gpps-ps*, PlacIq-*lacIq*	This work
pBBR-αGppsPs-Idi	Ptrc:*lacO*-RBSα-*gpps-ps*,Ptac:*lacO*-*idi*, PlacIq-*lacIq*	This work
pBBR-αGppsPs-Dxs	Ptrc:*lacO*-RBSα-*gpps-ps*,, Ptac:*lacO-dxs*, PlacIq-*lacIq*	This work
pBBR-αGppsPs-Dxr	Ptrc:*lacO*-RBSα-*gpps-ps*,, Ptac:*lacO-dxr*, PlacIq-*lacIq*	This work
pBBR-αGppsPs-IspD	Ptrc:*lacO*-RBSα-*gpps-ps*,, Ptac:*lacO-ispD*, PlacIq-*lacIq*	This work
pBBR-αGppsPs-IspE	Ptrc:*lacO*-RBSα-*gpps-ps*,, Ptac:*lacO-ispE*, PlacIq-*lacIq*	This work
pBBR-αGppsPs-IspF	Ptrc:*lacO*-RBSα-*gpps-ps*,, Ptac:*lacO-ispF*, PlacIq-*lacIq*	This work
pBBR-αGppsPs-IspG	Ptrc:*lacO*-RBSα-*gpps-ps*,, Ptac:*lacO-ispG*, PlacIq-*lacIq*	This work
pBBR-αGppsPs-IspH	Ptrc:*lacO*-RBSα-*gpps-ps*,, Ptac:*lacO-ispH*, PlacIq-*lacIq*	This work
pBBR-αGppsPs-IdiDxs	Ptrc:*lacO*-RBSα-*gpps-ps*,, Ptac:*lacO-idi dxs*, PlacIq-*lacIq*	This work
pBBR-αGppsPs-IdiDxsDxr	Ptrc:*lacO*-RBSα-*gpps-ps*,, Ptac:*lacO-idi dxs dxr*, PlacIq-*lacIq*	This work
pBBR-βGppsPs	Ptrc:*lacO*-RBSβ-*gpps-ps*, PlacIq-*lacIq*	This work
pBBR-γGppsPs	Ptrc:*lacO*-RBSγ-*gpps-ps*, PlacIq-*lacIq*	This work
pBBR-γGppsPs-IdiDxs	Ptrc:*lacO*-RBSγ-*gpps-ps*, Ptac:*lacO-idi dxs*, PlacIq-*lacIq*	This work
pBBR-γGppsPs-IdiDxsDxr	Ptrc:*lacO*-RBSγ-*gpps-ps*, Ptac:*lacO-idi dxs dxr*, PlacIq-*lacIq*	This work
pBBR-αGFP	Ptrc:*lacO*-RBSα-*gfp*, PlacIq-*lacIq*	This work
pBBR-βGFP	Ptrc:*lacO*-RBSβ-*gfp*, PlacIq-*lacIq*	This work
pBBR-γGFP	Ptrc:*lacO*-RBSγ-*gfp*, PlacIq-*lacIq*	This work
RBSα (BBa_J95021)	GAGCAGAGGAGA	Registry of Standard Biological Parts
RBSβ (BBa_J95016)	CCTGGGGGAGGG	Registry of Standard Biological Parts
RBSγ (BBa_J95015)	CATCAACGGAGGT	Registry of Standard Biological Parts

### Transformation of plasmids into* R. sphaeroids*

The plasmids for pinene production were transferred into competent *E.coli* S17-1, respectively, in advance. Then, the corresponding plasmid was transferred from donor strain *E.coli* S17-1 into recipient strain *R. sphaeroids* 2.4.1 before pinene production using di-parental conjugation. 1 mL of *E.coli* S17-1 and *R. sphaeroids* 2.4.1 were collected at logarithmic growth phase and washed twice with the modified Sistrom's medium, respectively. Then the cells of *E.coli* S17-1 and *R. sphaeroids* 2.4.1 were resuspended in 300 and 500 µL of modified Sistrom's medium, respectively. 100 µL of *R. sphaeroids* 2.4.1 and 50 µL of *E.coli* S17-1 were mixed and spotted in the middle of a agar plate with modified Sistrom's medium. After 24 h, the mixed bacteria were collected from the plate, washed three times and then spread on agar plates with modified Sistrom's medium and kanamycin. Plates were incubated at 30 °C until new colonies appeared. The transformants were screened and identified through colony PCR with the primers 5′-CCTGTGCCATCGAAATGA TCCACACCAT-3′ and 5′-AGTTCGTCGCTCGACTTC GTGACATCAA-3′.

### Pinene production and analytical methods

*R. sphaeroids* 2.4.1 transformants carrying the proper plasmids were cultured and harvested at logarithmic growth phase and then inoculated into the pinene producing medium with initial OD_660_ of 0.03–0.04. All the experiments were carried out in sealed transparent bottles with the volume of 155 mL filled with culture medium. Dodecane with the content of 1.2% (v/v) and IPTG with proper concentration were added to the culture when cells reached an OD_660_ of 0.3–0.4. Each test was conducted in triplicate. Cells were cultured in a closed light incubator without agitation. Bottles were turned upside down 5 times every eight hours, just in case bacterial precipitation occurred. After 132 hs’ cultivation, dodecane were collected and centrifuged, and then mixed at the ratio of 1:1(v/v) with limonene/ethyl acetate mixture in which the concentration of limonene was 6 mg/L. The concentration of pinene was tested with limonene as an internal standard by GC–MS (Agilent 7890A with Agilent 5975 MS detector) using the method proposed by Sarria et al. [6]. The absorbance of bacteria at 660 nm with the volume of 200 µL was measured by Multiskan FC (Thermo Scientific) plate-reader. And dry cell weight was calculated as follow:

1 $$Y=1.3731X$$

where X was the value of OD_660_ and Y was dry cell weight (mg/mL). And the correlation coefficient value (*R*^2^) was 0.9921.

### Measurement of fluorescence intensity

After growing for 72 h under the conditions of pinene production, 1000 µL of bacteria culture was taken from the bottles and centrifuged at 6000 rpm for 5 min. Pellets were then washed for three times and resuspend with 1000 µL of PBS buffer. 100 µL of the resuspend bacteria was added into 96-well plates. Each sample was tested in triplicate. Using PBS buffer as blank, the fluorescence intensity was measured by Fluoroskan Ascent FL (Thermo Scientific) plate-reader at an excitation wavelength of 492 nm and an emission wavelength of 518 nm.

## Supplementary Information

**Additional file 1: Fig. S1.** The growth curve of *R. sphaeroides* harboring pBBR-αGppsPs plasmid at various IPTG concentrations.

## Data Availability

The datasets for plasmids construction are available from Mendeley Data (http://dx.doi.org/10.17632/m7r93c3tsh.1). All other data generated or analyzed during this study are included in this published article and its additional file.

## Abbreviations

GPPS: Geranyl diphosphate synthase
PS: Pinene synthase
IPP: Isopentenyl pyrophosphate
DMAPP: Dimethylallyl pyrophosphate
MEP: 2C-methyl-d-erythritol 4-phosphate
GPP: Geranyl pyrophosphate
RBS: Ribosome binding site
CrtE: Prenyltransferase
FPP: Farnesyl pyrophosphate
Pyr: Pyruvate
G3P: Glyceraldehyde-3-phosphate
DXP: 1-Deoxy-d-xylulose 5-phosphate
Dxs: 1-Deoxy-d-xylulose 5-phosphate synthase
Dxr: 1-Deoxy-d-xylulose 5-phosphate reductase
IspD: 2C-methyl-d-erythritol 4-phosphate cytidylyltransferase
IspE: 4-Diphosphocytidyl-2C-methyl-d-erythritol kinase
IspF: 2C-methyl-d-erythritol 2,4-cyclodiphosphate synthase
IspG: 2C-methyl-d-erythritol 2,4-cyclodiphosphate reductase
IspH: 4-Hydroxyl-3-methylbut-2-enyl diphosphate reductase
Idi: Isopentenyl diphosphate isomerase
AcCoA: Acetyl-CoA
AACT: Acetoacetyl-CoA thiolase
HMGS: 3-Hydroxy-3-methyl-glutaryl-CoA synthase
HMGR: 3-Hydroxy-3-methyl-glutaryl-CoA reductase
MK: Mevalonate kinase
PMK: Phosphomevalonate kinase
PMD: Diphosphomevalonate decarboxylase
