# Supplementary material for: Biosynthesis of pinene in purple non-sulfur photosynthetic bacteria
Source: Microb Cell Fact. 2021 May 17;20:101. doi: 10.1186/s12934-021-01591-6 (PMC8130110; doi:10.1186/s12934-021-01591-6)
Supplement: Supplementary file 1 — Additional file 1: Fig. S1. The growth curve of R. sphaeroides harboring pBBR-αGppsPs plasmid at various IPTG concentrations. [file 12934_2021_1591_MOESM1_ESM.docx]

Fig. S1 The growth curve of *R. sphaeroides* harboring pBBR-αGppsPs plasmid at various IPTG concentrations. The initial dry cell weight was 0.147 mg/mL and IPTG was added at the beginning. Errors indicate s.d. (n = 3).
